# Supplementary material for: Akkermansia muciniphila mediates negative effects of IFNγ on glucose metabolism
Source: Nat Commun. 2016 Nov 14;7:13329. doi: 10.1038/ncomms13329 (PMC5114536; doi:10.1038/ncomms13329)
Supplement: Supplementary Information — Supplementary Figures 1-6 and Supplementary Tables 1-2. [file ncomms13329-s1.pdf]

Supplementary Materials

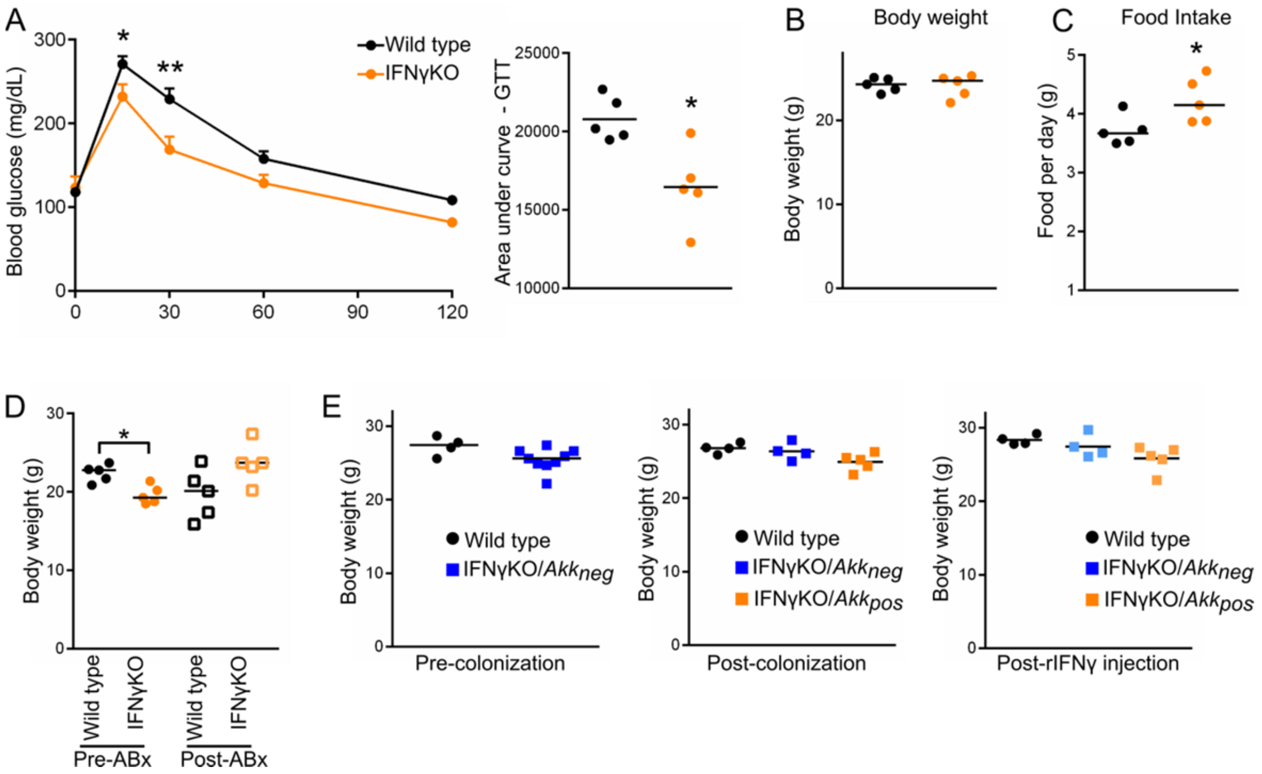

Supplementary Figure 1: Improved glucose tolerance in IFNγKO mice is not explained by reduced weight or food intake

(A-C) Measurement of glucose tolerance (A)**,** body weight (B), and food intake per day (C) in a cohort of wild type and IFNγKO mice. **(D)** Body weight measurements for groups analyzed in Fig. 1A. **(E)** Body weight measurements for glucose tolerance experiments shown in Fig. 3. Glucose tolerance curves shown as mean ± SEM. Median line is displayed on dot plots. n=5 per group for (A-D). n=5 per group or as indicated by individual points. *$p<0.05$, **$p<0.01$ by one-tailed Mann-Whitney test.

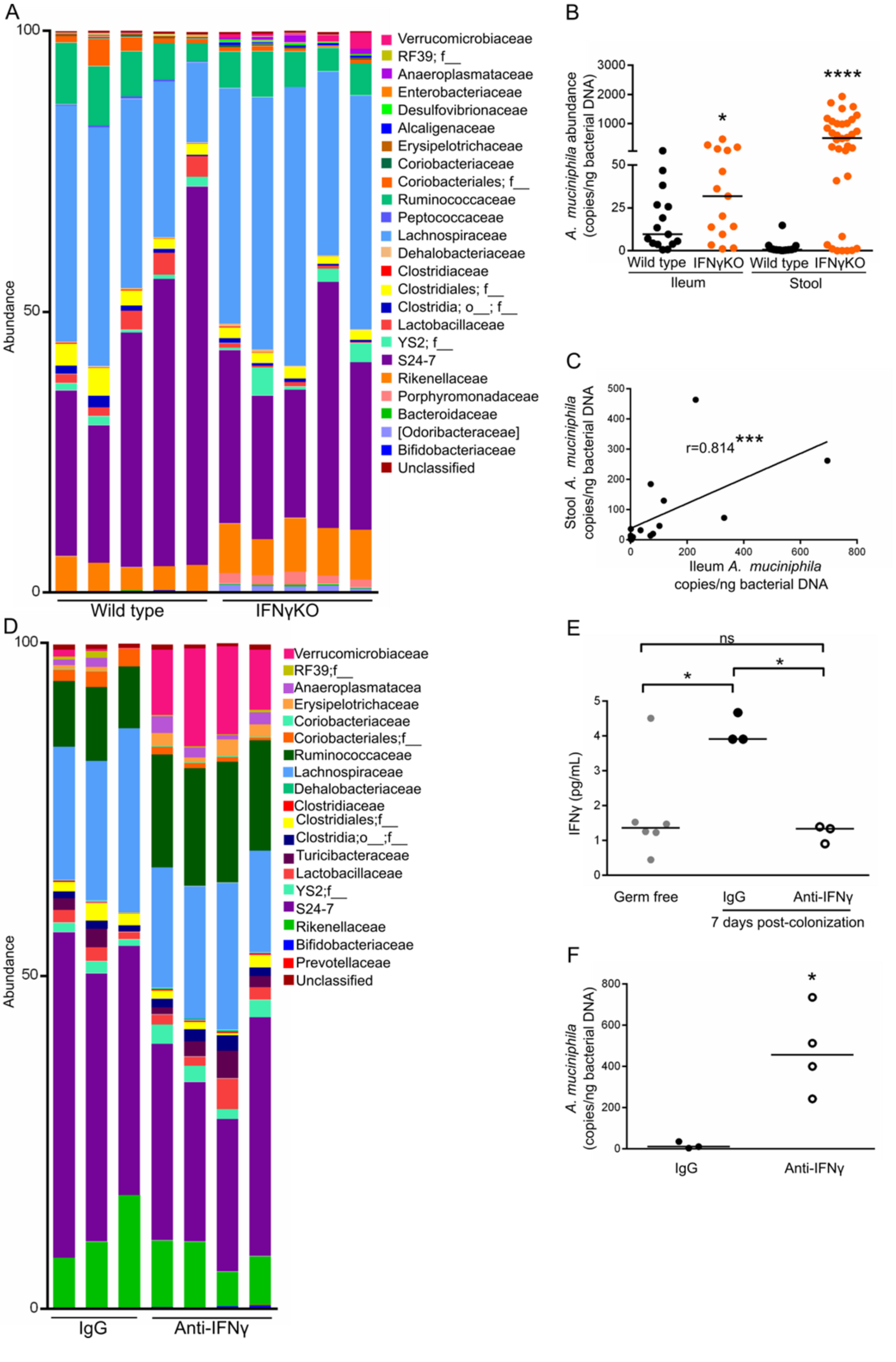
A
B
C
D
E
F
Verrucomicrobiaceae
RF39; f__
Anaeroplasmataceae
Enterobacteriaceae
Desulfovibrionaceae
Alcaligenaceae
Erysipelotrichaceae
Coriobacteriaceae
Coriobacteriales; f__
Ruminococcaceae
Peptococcaceae
Lachnospiraceae
Dehalobacteriaceae
Clostridiaceae
Clostridiales; f__
Clostridia; o__; f__
Lactobacillaceae
YS2; f__
S24-7
Rikenellaceae
Porphyromonadaceae
Bacteroidaceae
[Odoribacteraceae]
Bifidobacteriaceae
Unclassified
Abundance
Wild type
IFNγKO
A. muciniphila abundance (copies/ng bacterial DNA)
Ileum
Stool
Stool A. muciniphila copies/ng bacterial DNA
r=0.814***
Ileum A. muciniphila copies/ng bacterial DNA
Anaeroplasmatacea
Turicibacteraceae
Prevotellaceae
IgG
Anti-IFNγ
IFNγ (pg/mL)
ns
Germ free
7 days post-colonization
A. muciniphila (copies/ng bacterial DNA)

Supplementary Figure 2: Altered microbiota composition in IFNγKO mice and by IFNγ neutralization during colonization

(A) Relative abundances (percent of total 16S gene sequences) of microbial families in representative samples of wild type and IFNγKO mice. Each bar represents one individual. Where family assignment could not be made, the most specific level of assignment available is provided. **(B)** *A. muciniphila* quantification in whole ileum and stool by qPCR in wild type and IFNγKO mice. **(C)** Spearman correlation of *A. muciniphila* in stool to *A. muciniphila* in whole ileum represented as copies *A. muciniphila* genome/ng total 16S DNA. **(D)** Relative abundances (percent of total 16S gene sequences) of microbial families in representative samples of IgG and anti-IFNγ treatment seven days after colonization with microbes. Where family assignment could not be made, the most specific level of assignment available is provided. Each bar represents one individual. **(E)** Serum levels of IFNγ in germ free mice and during colonization of germ free mice with and without neutralization of IFNγ by antibody injection. **(F)** *A. muciniphila* abundance in the cecum by in anti-IFNγ and IgG control treatments, represented as copies *A. muciniphila* genome/ng total 16S DNA determined by qPCR. All individuals are shown, median line is displayed on dot plots.*$p<0.05$, ***$p<0.001$, **** $p<0.0001$ by one-tailed Mann-Whitney test.

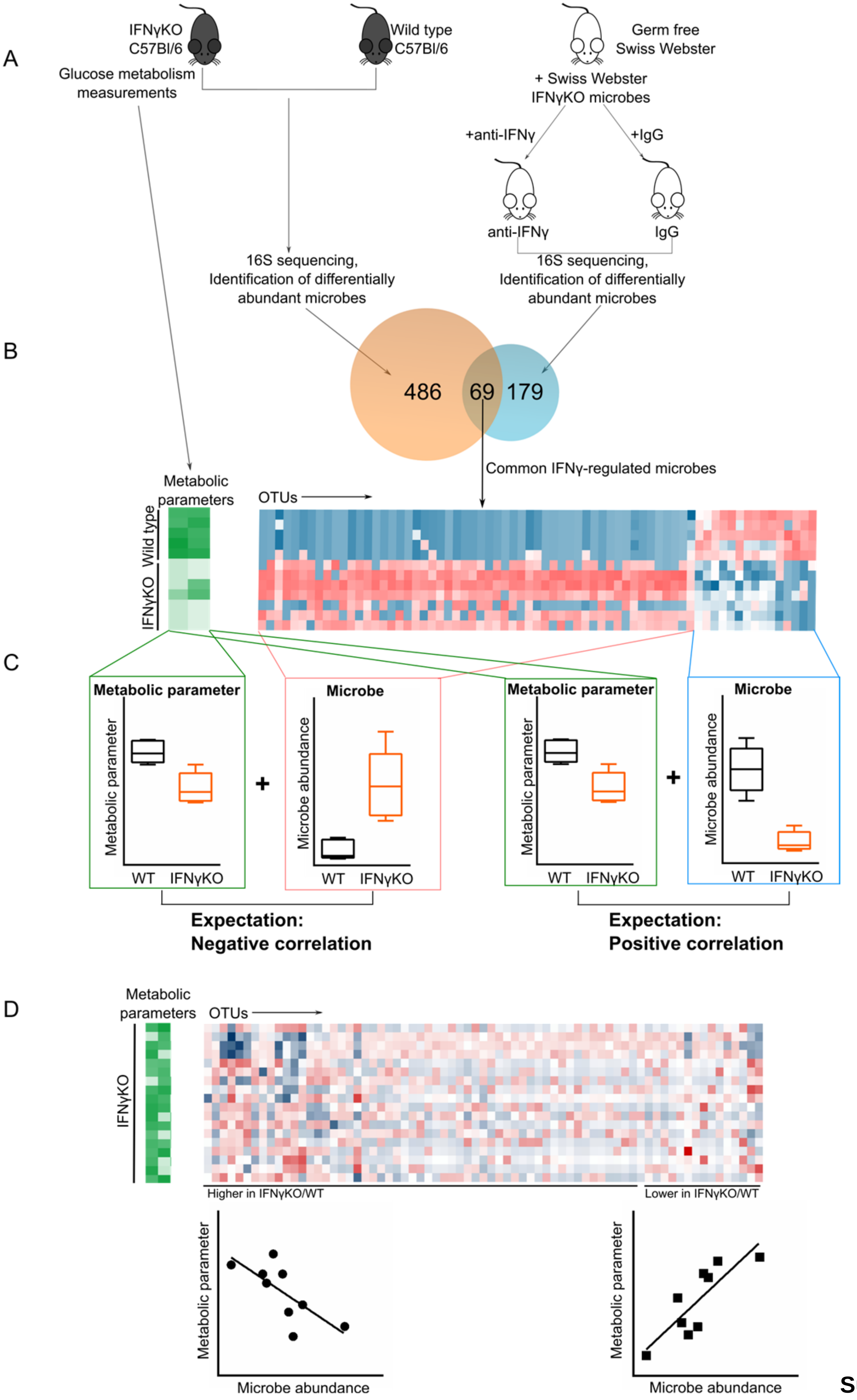

Supplementary

Figure 3: Scheme of experiments and analyses for causal inference of IFNγ-regulated microbial regulators of glucose tolerance

To identify candidate microbes for regulation of the effect of IFNγ on glucose metabolism we employed a strategy based on causal inference as outlined here using representative data. **(A)** Two independent experimental perturbations of IFNγ levels were employed – genetic knockout and neutralizing antibody treatment. **(B)** Microbiota composition of each comparison described in (A) was determined by 16S rRNA sequencing and differentially abundance microbes within each perturbation experiment were identified. From these microbes, those in common to both experimental strategies were considered for further analysis, as they demonstrate consistent regulation by IFNγ. **(C)** Given that glucose metabolism parameters show a positive correlation with IFNγ presence (i.e. fasting glucose and glucose tolerance measurements are higher in wild type and IgG comparing to IFNγKO and anti-IFNγ, respectively, as shown in (B)), we can establish an expected relationship between each OTU and metabolic parameters. Specifically, we expect that microbes that are higher in the absence of IFNγ will have a negative correlation with metabolic parameters (high microbe abundance in the context of low metabolic measures). Correspondingly, OTUs that have lower abundance in the absence of IFNγ are expected to have a positive correlation with metabolic parameters if they are true candidates for metabolic regulators. **(D)** To identify correlations, we utilized natural variation in both OTU abundance and metabolic parameter measurements within IFNγKO mice. Representative data for metabolic parameters and OTU abundance has now been scaled only on IFNγKO mice to demonstrate intergroup variability. Those OTUs that do not match expectations as laid out in (C) are eliminated as possible candidates. In this manner we more precisely identify predicted IFNγ-dependent microbial regulators of glucose metabolism.

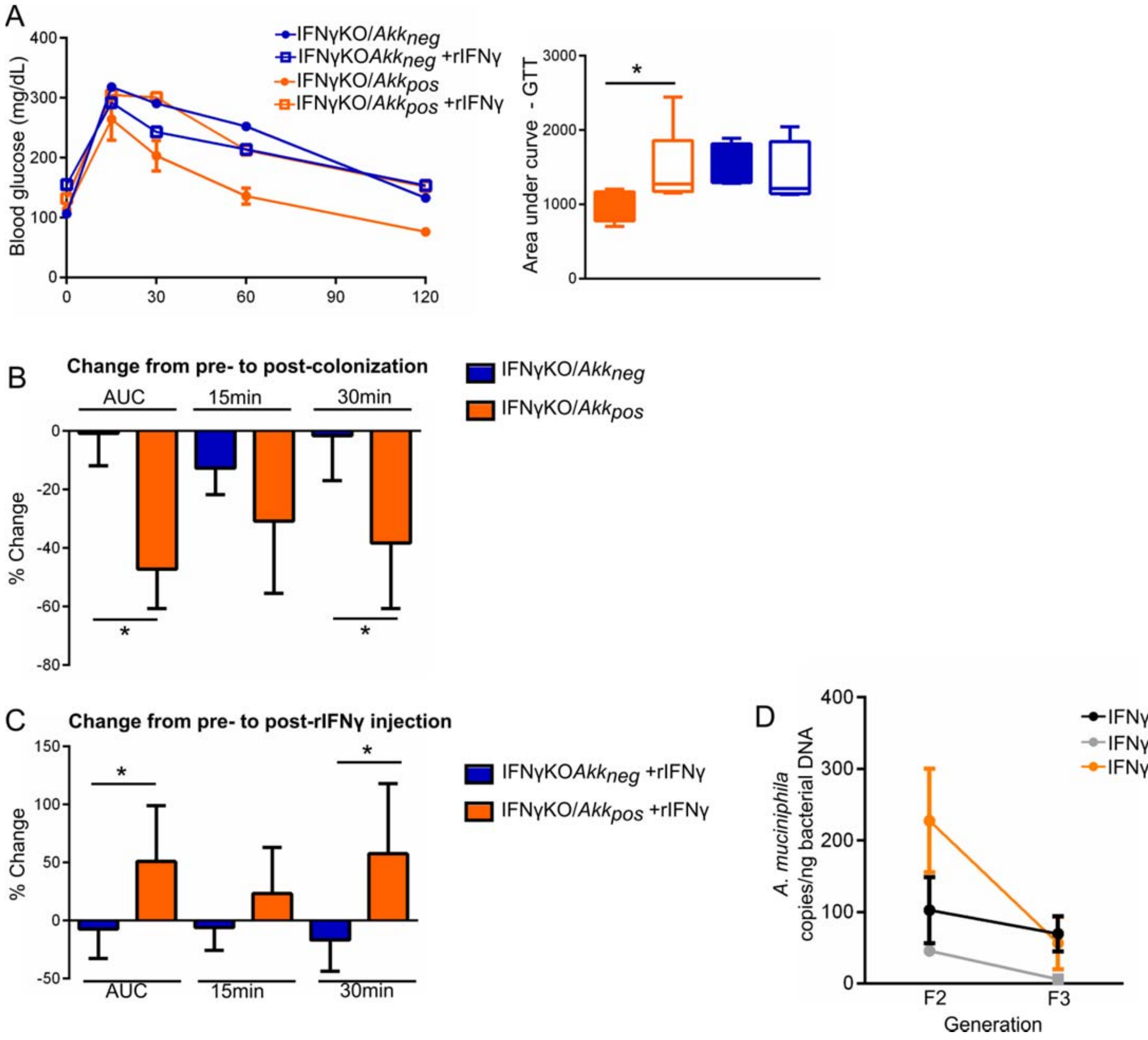

Supplementary Figure 4: Worsening of glucose tolerance by IFNγ injection requires *A. muciniphila*

Additional analysis of experiments described in Fig. 2, showing comparison of IP-GTT in IFNγ/Akk_{pos} and IFNγ/Akk_{neg} mice before and after two weeks of injection of rIFNγ. **(A)** Glucose tolerance test pre- and post-rIFNγ injection showing comparison of all groups. Solid symbols represent post-colonization (pre-injection) time points and open symbols represent post-rIFNγ injection. IFNγ/Akk_{neg} mice are in blue and IFNγ/Akk_{pos} mice are in orange. **(B)** Percent change of AUC-GTT, 15 minute and 30 minute glucose measures between pre-colonization and post-colonization time point in IFNγ/Akk_{pos} and IFNγ/Akk_{neg} mice. **(C)** Percent change of AUC-GTT, 15 minute and 30 minute glucose measure between post-colonization time point and post-rIFNγ injection time point in IFNγ/Akk_{pos} and IFNγ/Akk_{neg} mice. **(D)** Levels of *A. muciniphila* in stool, assessed by qPCR over two generations of heterozygous interbreeding. F0 breeding of IFNγKO to C57Bl/6 wild type was used to generation heterozygous F1 offspring. These heterozygous F1 mice were then interbred to generate wild type, heterozygous and knockout offspring (F2), shown here. Heterozygous F2 offspring were again interbred to generate F3. Within two generations of IFNγ heterozygous interbreeding, *A. muciniphila* levels are normalized across genotypes, presumably due to absence of initial exposure from parents. Glucose tolerance curves shown as mean ± SEM, box plots represent median with 25th and 75th percentile borders and error bars represent min-max, bar charts are mean + SD, n=4 (IFNγKO/Akk_{neg}) or 5 (IFNγKO/$Akk_{pos.}$), *p<0.05 by one-tailed Mann-Whitney test.

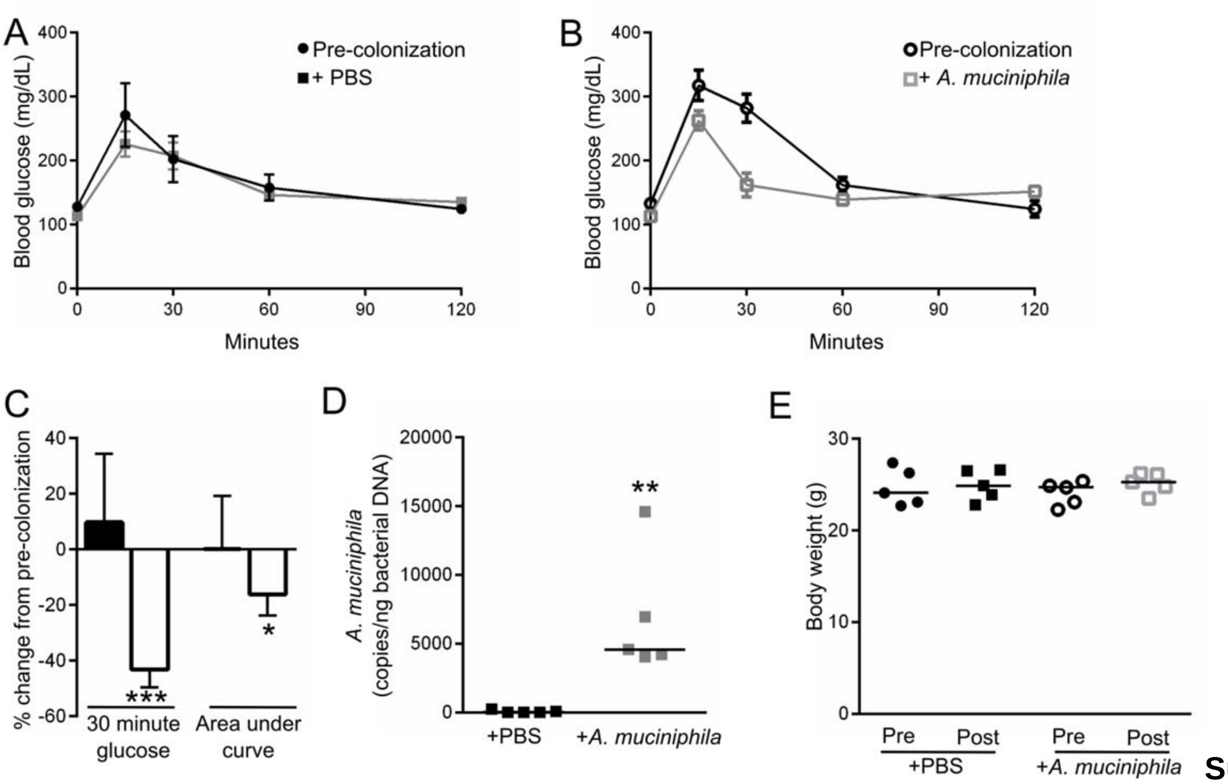

Supplementary

Figure 5: *A. muciniphila* improves glucose tolerance in lean wild type mice

(A-B) Glucose tolerance in lean wild type mice before (day 0) and after (day 14) mock colonization (A) or colonization with *A. muciniphila* shows that mock colonization mice maintain glucose tolerance levels, while mice given *A. muciniphila* (B) improve glucose tolerance two weeks post-colonization. **(C)** Quantification of percent change of area under the curve of the glucose tolerance test and 30 minute blood glucose from pre- to post-colonization time point for each colonization group. **(D)** *A. muciniphila* abundance (copies/ng total 16S DNA) by qPCR at day 14 post colonization. **(E)** Body weights of each group pre- and post-colonization. N=5 per group. Glucose tolerance curves shown as mean ± SEM, bar charts are mean + SD, and dot plots display median line. *p<0.05, **p<0.01, ***p<0.001 by one-tailed Mann-Whitney test.

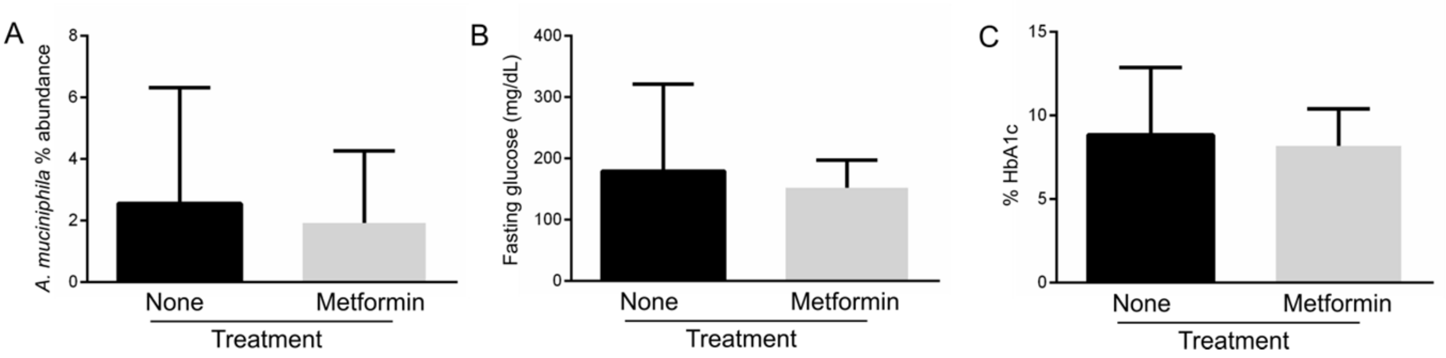

Supplementary Figure 6: Metformin treatment does not affect levels of *A. muciniphila*

A. muciniphila percent abundance (A), fasting glucose (B) and % HbA1c (C) in untreated (n=5) and metformin treated (n=6) type 2 diabetic subjects. Bar charts represent mean + 95% confidence intervals.

Supplementary Table 1: Differentially abundant microbes in breeding-derived IFNγKO/*Akk*$_{neg}$ versus IFNγKO/native *Akk*$_{pos}$

Parametric p-value	FDR	IFNγKO *Akk*$_{neg}$	IFNγKO native *Akk*$_{pos}$	Fold-change	UniqueID
< 1e-07	< 1e-07	1.30E-05	0.01	0.0012	k__Bacteria;p__Verrucomicrobia;c__Verrucomicrobiae; o__Verrucomicrobiales;f__Verrucomicrobiaceae;g__Akkermansia
1.04E-05	0.00241	1.20E-05	0.00063	0.02	k__Bacteria;p__Firmicutes;c__Clostridia;o__Clostridiales;f__Peptostreptococcaceae;g__
5.26E-05	0.00812	0.016	0.0073	2.23	k__Bacteria;p__Firmicutes;c__Clostridia;o__Clostridiales;f__;g__
0.0003547	0.0411	0.0012	1.30E-05	96.04	k__Bacteria;p__Proteobacteria;c__Betaproteobacteria;o__Burkholderiales;f__Alcaligenaceae;g__Sutterella

Supplementary Table 2: Differentially abundant microbes upon *A. muciniphila* colonization

	Correlation with GTT_ AUC		pre versus post colonization				
**UniqueID**	**Correlation coefficient**	**p-value**	**p-value**	**FDR**	**Abundance pre-colonization**	**Abundance in post-colonization**	**Akk v.s. noAkk Fold-change**
k__Bacteria;p__Firmicutes; c__Bacilli;o__Bacillales;f__ Staphylococcaceae;g__Sta phylococcus	-0.571	0.1943	1.20E-06	0.000556	0.0019	1.30E-05	145.8
k__Bacteria;p__Proteobact eria;c__Betaproteobacteria ;o__Burkholderial es;f__Alc aligenaceae;g__Sutterella	-0.333	0.3847	6.54E-05	0.0151	0.0015	0.00036	4.26
k__Bacteria;p__Verrucomi crobia;c__Verrucomicrobia e;o__Verrucomicrobiales;f_ _Verrucomicrobiaceae;g__ Akkermansia	-0.771	0.1027	0.0004 574	0.0706	0.00052	9.00E-06	57.54
